# The role of mechanical forces in the planar-to-bulk transition in growing *Escherichia coli* microcolonies

**DOI:** 10.1098/rsif.2014.0400

**Published:** 2014-08-06

**Authors:** Matthew A. A. Grant, Bartłomiej Wacław, Rosalind J. Allen, Pietro Cicuta

**Affiliations:** 1 Cavendish Laboratory, University of Cambridge, JJ Thomson Avenue, Cambridge CB3 0HE, UK; 2 SUPA, School of Physics and Astronomy, University of Edinburgh, James Clerk Maxwell Building, King's Buildings, Mayfield Road, Edinburgh EH9 3JZ, UK

**Keywords:** bacterial microcolony, bacterial biofilm, mechanics, cell growth

## Abstract

Mechanical forces are obviously important in the assembly of three-dimensional multicellular structures, but their detailed role is often unclear. We have used growing microcolonies of the bacterium *Escherichia coli* to investigate the role of mechanical forces in the transition from two-dimensional growth (on the interface between a hard surface and a soft agarose pad) to three-dimensional growth (invasion of the agarose). We measure the position within the colony where the invasion transition happens, the cell density within the colony and the colony size at the transition as functions of the concentration of the agarose. We use a phenomenological theory, combined with individual-based computer simulations, to show how mechanical forces acting between the bacterial cells, and between the bacteria and the surrounding matrix, lead to the complex phenomena observed in our experiments—in particular the observation that agarose concentration non-trivially affects the colony size at transition. Matching these approaches leads to a prediction for how the friction between the bacteria and the agarose should vary with agarose concentration. Our experimental conditions mimic numerous clinical and environmental scenarios in which bacteria invade soft matrices, as well as shedding more general light on the transition between two- and three-dimensional growth in multicellular assemblies.

## 1. Introduction

The assembly of three-dimensional multicellular structures is a central theme in biology, from embryology to cancer [1,2]. In general, this process is believed to be controlled by an interplay between mechanical forces between cells and their surrounding matrix [3,4], regulated cell growth (death and differentiation) [5–7], biochemical interactions between cells [8] and with the matrix [9] and cell migration [10]. However, dissecting the individual roles played by each of these factors remains largely an open challenge. Even from a purely mechanical point of view, our understanding of the forces exerted between cells, and between cells and their environment, remains limited.

An important stage in many multicellular developmental processes is the transition from two-dimensional to three-dimensional growth [1]. Examples include eye formation in vertebrates, which begins as a ‘bulge’ protruding from the surface of the ventrolateral forebrain [11], and invasion of neighbouring tissues by skin cancer cells, initially confined to two dimensions by the basal membrane [2]. For some bacteria, an important transition from two- to three-dimensional growth occurs in biofilms which form on surfaces such as water pipes [12], or teeth as well as in softer environments such as the skin [13] or in foods [14], and which are implicated in a variety of human pathologies [15]. In the laboratory, bacterial microcolonies are often grown trapped between a soft agarose pad and a glass slide, in order to investigate gene regulatory and physiological processes at the single-cell level [16]. In these experiments, even for bacterial strains that do not form biofilms with extracellular matrices, cells initially grow in two dimensions, but eventually invade the agarose to form multiple layers, hindering the tracking of individual cells. It is likely that a similar transition occurs in clinically relevant bacterial biofilms growing at the interface between hard and soft materials such as surgical implants or catheters surrounded by tissue [17].

**Figure 1.**
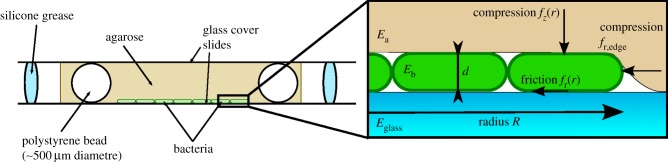
Experimental set-up. A thin slab of LB–agarose is confined between two microscope slides. The agarose contains a small number of polystyrene beads which act as spacers and ensure that the thickness of the agarose slab is constant at 500 μm. The bacteria are pipetted onto the top of the agarose before it is covered with the upper glass slide, and silicone grease is applied around the agarose to seal the sample. Zoomed-in region: illustration of the forces acting on the colony in our simulations. The microcolony is modelled as a flat disc of fixed height *d* and variable radius *R*, which is compressed vertically (force per unit colony area *f_z_*(*r*)) and radially (force per unit boundary area *f*_r*,*edge_) by the agarose, and radially by friction between the cells and the agarose (force per unit colony area *f*_r_(*r*)). (Online version in colour.)

In this work, we investigate the role of mechanical forces in the transition from two- to three-dimensional growth for *Escherichia coli* bacteria sandwiched between a glass slide and an agarose gel (figure 1), using experiments, phenomenological theory and computer simulations. Under these conditions, bacteria proliferate to form microcolonies, which are initially confined to the surface of the agarose, but eventually invade the agarose to form a three-dimensional community. In these microcolonies, the cells do not demonstrate any active motility (such as swimming, swarming or gliding [18,19]), but they do move owing to ‘pushing’ interactions with other cells as they proliferate. This system provides us a useful simple model for three-dimensional multicellular assembly, because relatively few factors are at play. We show that the elasticity of the substrate and of the bacteria (which are slightly compressed and bent by the interaction with the substrate) plays an important and non-trivial role in determining the colony size at which this transition happens. Our results can be explained purely by mechanical forces, with some simple assumptions about the nature of the friction between the bacteria and the agarose. Matching our simulations to our experimental data leads to predictions for the dependence of these forces on the agarose concentration. More generally, our work should lead to better understanding of the invasion of soft materials by bacteria, and shed light on how mechanical interactions between cells and their environment can lead to the emergence of complex three-dimensional structures.

## 2. Material and methods

### 2.1. Experimental methods

#### 2.1.1. Agarose preparation

Agarose (Sigma-Aldrich, A9539) was mixed with Luria Bertani (LB) broth powder (Sigma-Aldrich, L3022, containing 10 g l^−1^ tryptone, 5 g l^−1^ yeast extract and 5 g l^−1^ NaCl) and water (to make a final LB powder concentration of 2 wt% and then autoclaved). This concentration of LB broth powder was chosen to maximize the growth rate of *E. coli*. Once the autoclaving cycle was complete, the agarose–LB mix was stirred for 1 h at 80°C, to ensure thorough mixing, and then stored at a temperature (≈50°C) that kept the solution in a liquid form. A range of different concentrations of agarose were explored, from 1.5 to 4 wt%: this is the widest possible range in these experiments, because, less than 1.5%, the agarose becomes too soft and the bacteria become motile, whereas, more than 4 wt%, the very high viscosity prevents sample preparation.

#### 2.1.2. Bacterial strain and growth conditions

The fluorescently tagged *E. coli* K-12 strain BW25113 + PKK_PdnaA-GFP (which contains the gene for GFP under the control of the *dnaA* promoter on a plasmid) was grown overnight in 5 ml of LB supplemented with 5 µl of 100 mg ml^−1^ ampicillin (to make a final concentration 100 µg ml^−1^) at 37°C. The overnight culture was diluted into fresh LB at a ratio 1 : 300, 1 h before use; the bacteria were in exponential phase when the experiment commenced.

#### 2.1.3. Sample preparation

Trace amounts of polystyrene beads of diameter 500 µm were mixed with the liquid agarose in an Eppendorf tube. One millilitre of this agarose–bead mixture was then pipetted onto a cover slide, compressed with a second cover slide and allowed to dry. A small section of this agarose was then cut and placed on a new microscope cover slide. The bacterial culture (2 µl; in exponential phase) was then pipetted onto the agarose in a single drop. The slide around the agarose was covered in silicone grease (to seal the sample), and a cover slide was placed on top. The sparse 500 µm beads act as spacers and ensure that the agarose layer between the coverslip and microscope slide remains of constant depth during the imaging process.

#### 2.1.4. Data collection

**Figure 2.**
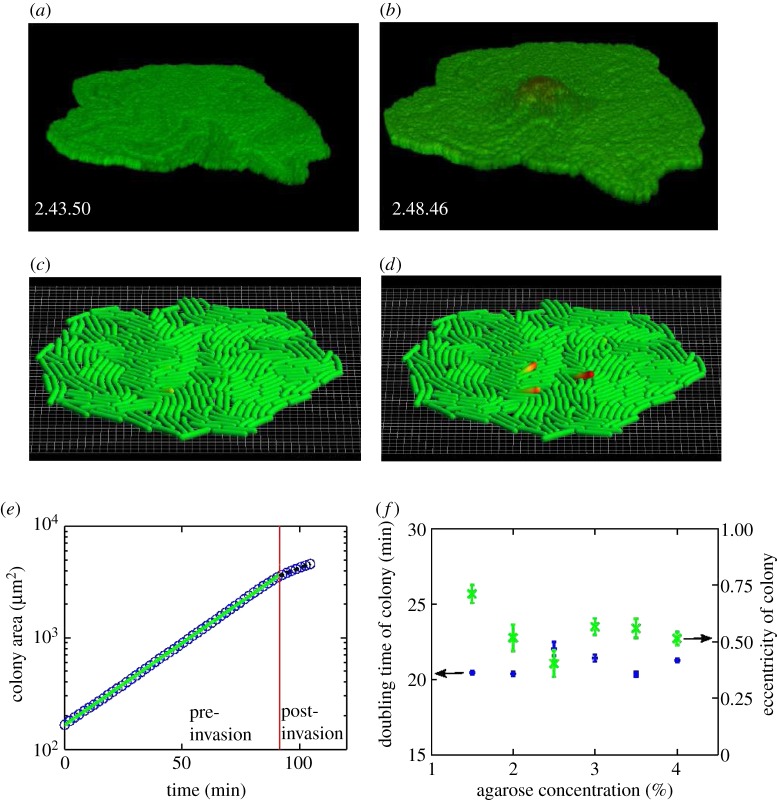
The transition from two- to three-dimensional growth in *E. coli* microcolonies. (*a,b*) Confocal microscopy images from our experiments of a microcolony just before (*a*) and after (*b*) the invasion; the microcolony is shaded for depth, showing in dark where invasion has occurred. ‘Up’ in these images is the direction towards the agarose, consistent with the sketch of figure 1. (*c,d*) Simulations of a microcolony show the same phenomenon: snapshots of the *in silico* colony are shown before (*c*) and after (*d*) invasion; bacteria are shown in red if they have invaded the agarose. (*e*) An example of our experimental data for microcolony area as a function of time: the area first increases exponentially (straight segment of the plot), but upon invasion the observed area growth rate decreases. The solid line shows the best fit to the initial exponential growth phase, whereas the dashed line shows an exponential fit to the ‘post-invasion’ phase. The red vertical line indicates the invasion time, as determined by the best fit for the intersection of the solid and dashed lines (i.e. fitting with the two segments with a free crossover point). (*f*) The average microcolony doubling time (circles; left axis), obtained from the pre-invasion area growth rate, is unaffected by agarose concentration. The crosses (right axis) show the average eccentricity of microcolonies at buckling, showing that the colonies are fairly circular. Error bars are standard errors on the mean.

The sample was imaged using a Leica SP5 confocal microscope, with an automated stage allowing for several microcolonies to be tracked over time. To obtain the area of the microcolony (which was measured at intervals of 1 min), the microscope's transmitted beam was recorded (equivalent to bright field imaging). In some experiments, to obtain three-dimensional images of the microcolony, the microscope was used in confocal mode in order to build up a *z*-stack (figure 2*a,b*). All experiments were performed at 37°C.

#### 2.1.5. Data analysis

Each bright field image was analysed using custom scripts written in Matlab: a simple intensity threshold on the image was sufficient to distinguish the colony from the background, and hence the area and shape could be calculated; watershed filtering was used to segment individual cells in selected experiments.

#### 2.1.6. Detection of the invasion transition

The time at which the microcolony invaded the agarose was identified by finding the discontinuity in its area growth rate (figure 2*e*), which was found (from our confocal images) to correspond to the moment at which the first cell escapes from the two-dimensional layer. The invasion transition can also be identified by eye in our brightfield images, but in these images a small shift in focus can prevent the first invading cell from being spotted immediately. Use of confocal imaging in all our experiments, while accurate, would have been much more resource intensive.

### 2.2. Simulations

#### 2.2.1. Individual-based model for elastic bacteria

Our computer simulations were implemented in a purpose-written C++ program. Individual bacteria were modelled as elastic rods, represented by *n*_spheres_ = 8*–*16 overlapping spheres (with new spheres added as the rod grows), linked together via nonlinear springs, such that the rod dynamics was described by Euler–Bernoulli dynamic beam theory [20]. Repulsive elastic interactions between bacterial cells were modelled by assuming that a repulsive force acts between spheres located in different bacterial rods. Because the exact nature of the elastic forces between bacterial cells is unknown, we used the generic functional form *F* = *CE*_b_(*d*/2)^2−*β*^*ε*^*β*^, where *F* is the magnitude of the repulsive force (acting along the line between the centres of the two spheres), *E*_b_ is the effective Young modulus of the bacteria, *d* is the diameter of the spheres (and hence of the bacteria), *ε* is the distance over which the two spheres overlap and the dimensionless coefficient *C* is of the order unity. The same expression was also used to represent the interaction between the cells and the solid glass surface, albeit with *E*_b_ replaced with the elastic modulus of glass *E*_g_. The choice of the exponent *β* allowed us to specify a particular contact force model. In most of our simulations, we assumed the classical Hertzian theory of contact mechanics [21], *F* = (4/3)*E*_b_(*d*/2)^1/2^*ε*^3/2^, so that *C* = 4/3 and *β* = 3/2. We found that our results were qualitatively the same for other choices of *β* (see appendix F). It is important to note that the effective Young modulus *E*_b_ encapsulates the elastic properties of the whole bacterial cell (i.e. cell wall and cytoplasm), and is therefore different to the elastic modulus that might be measured for the cell wall only.

In addition to these repulsive forces, cells also experienced dry static frictional forces (according to Amonton's laws of friction, with friction coefficient 0.3); these were assumed to act between any pair of cells that were in contact. The frictional forces acted in the direction opposite to the local sliding velocity. In some simulations, we also used Stokes-like, velocity-dependent friction, as described in appendix E.

#### 2.2.2. Bacterial growth

Cell growth was modelled by a linear expansion of the rod length; upon reaching twice its initial length, each rod was split into two, producing two equal-sized daughter cells. We assumed the average length of an uncompressed bacterium to be 5 µm, in agreement with our experimental data for low agarose concentrations. We included stochasticity in the growth dynamics by allowing the growth rate of individual cells to vary by ±10% around a mean doubling time of 20 min (this meant that the simulation lost synchrony in cell division after about 10 generations, as has been observed experimentally [22]). Nutrients were not modelled explicitly (the mean growth rate was assumed to be the same for all cells).

#### 2.2.3. Interactions between bacteria and agarose

The agarose was modelled implicitly in our simulations, via vertical and horizontal compression forces, and horizontal frictional forces, acting on the bacteria. These forces were calculated by assuming that a bacterial microcolony behaves as a rigid, circular punch of radius *R*, obtained from the colony area *A* as 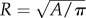. Vertical compression of the bacteria by the agarose was represented by a force of magnitude 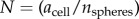 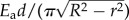, acting on each of the *n*_spheres_ spheres making up a bacterial rod (cf. equation (3.3)). Here, *a*_cell_ is the horizontal area of a given bacterium (which can change owing to growth or compression), *r* is its radial distance from the microcolony centre, *d* = 1.4 µm is the height of the microcolony (this is a typical cell diameter as measured in our experiments) and *E*_a_ is the Young elastic modulus of the agarose. To avoid unphysical divergence of the vertical compression force at the edges of the microcolony, we imposed a cut-off, such that if *r* > *R* − 1 μm, 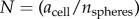 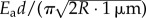.

Radial compression by the agarose was modelled by an inward radial force (*a*_cell_/*n*_spheres_)*E*_a_*d*/(2*πR*) acting on spheres that form part of bacteria located at the periphery of the microcolony. The theoretical arguments leading to these force functions are given in the Results section and in appendices A and G; in appendix C, we describe how they were modified in order to simulate pairs of colliding microcolonies.

In all our simulations, friction between the bacteria and the agarose, and between the bacteria and the glass surface, was represented by a force *F* = *kN* acting on each sphere in the direction opposite to its velocity, where *k* is the friction coefficient and *N* is the vertical compression force acting on that bacterium. We explored different functional forms for the dependence of the friction coefficient *k* on the concentration *C*_a_ of the agarose, as discussed in the Results section. In our simulations, we assumed static friction—i.e. that the frictional force does not depend on the velocity *v* of cells relative to the surface. This is different to previous works [23–25] which have assumed Stokesian-like friction proportional to the cells' velocity. In our opinion, the static friction model is more physically realistic; however, we show in appendix E that repeating our simulations with dynamic Stokesian friction *F* = *kvN* produces almost identical results.

#### 2.2.4. Elasticity parameters

The elastic modulus *E*_b_ of an *E. coli* cell in our experiments is not known (existing data vary between 0.1 and 200 MPa [26]); in our simulations, we found that *E*_b_ = 375 kPa gave a good fit to our experimental data. The elastic modulus of glass was set to *E*_glass_ = 10 MPa (i.e. 25 times larger than that of the bacteria); this value represents a compromise between computational speed and realism of the simulation.^1^ We varied the elastic modulus *E*_a_ of the agarose in the range 100–800 kPa, in agreement with the experimentally explored range of agarose concentrations.

#### 2.2.5. Triggering the two- to three-dimensional transition

In our growing colonies, we expect that the invasion transition happens when the ‘squeezing’ of the microcolony owing to radial (friction and/or compression) forces causes cells to move out of the horizontal plane, overcoming the vertical forces resulting from compression of the agarose. The translation of horizontal radial forces into vertical motion happens because of small local inhomogeneities in the surface and/or in the shape of individual bacteria as well as the Euler buckling instability in compressed rod-shaped bacteria. To reproduce this in our simulations, we introduced local inhomogeneities in the glass/agarose surfaces, represented by a sinusoidal modulation of the glass' height with 10 nm amplitude and period 1 µm along both horizontal axes (*x* and *y*).

#### 2.2.6. Dynamics

The dynamics of the system was modelled by solving Newton's equation of motion for the spheres, with the only source of damping coming from the frictional forces. We used a simple Euler method to integrate the system of differential equations for the position and velocity of each sphere with a fixed time step d*t* = 2*^−^*^18^ to 2*^−^*^16^ h.

#### 2.2.7. Detecting the two- to three-dimensional transition

In our simulations, the two- to three-dimensional transition happens very rapidly and can be detected accurately by measuring the vertical distance of the surface of each of the spheres in each bacterium from the glass surface (defined as the smallest distance between the surface of the sphere and the glass surface). The transition was defined to be the moment at which this distance was more than (1/2)*d* = 0.7 μm for any sphere.

## 3. Results

### 3.1. Experimental observations

#### 3.1.1. Bacterial microcolonies undergo a sharp ‘invasion’ transition from two- to three-dimensional growth

Tracking the microcolony area as a function of time in our experiments reveals that the colony grows exponentially throughout our experiments (suggesting that there is no nutrient limitation), but with a discontinuity in the area growth rate (figure 2*e*) which occurs after 80–90 min of growth for an agarose concentration of 3%. Confocal microscopy images (figure 2*a*,*b*) show that this discontinuity corresponds to the formation of a second layer of bacterial cells, i.e. the transition from two- to three-dimensional growth. In our experimental conditions, colonies grow at a constant rate independent of the agar concentration (figure 2*f*).

#### 3.1.2. Invasion of the agarose first occurs near the microcolony centre

**Figure 3.**
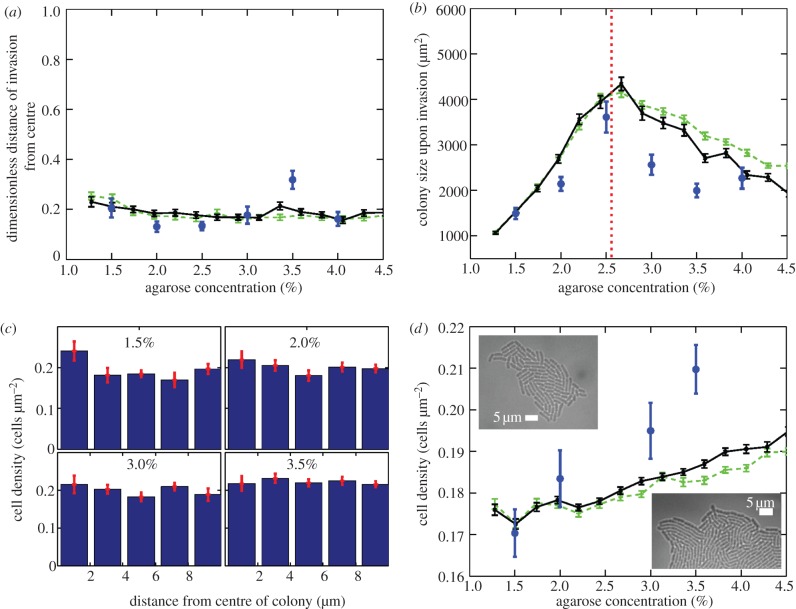
Simulations using only mechanical forces are able to match experimental observations. (*a*) The dimensionless distance of invasion from the centre of the colony. The circles show experimental data; markers connected by lines show simulation results where friction is given by equation (3.5) for *α* = 0.4 (dashed line) and *α* = 1 (solid line), with the friction coefficient from equation (3.8). (*b*) The area of the colony at which invasion first takes place. Data markers are the same as in (*a*). The vertical line marks the agarose concentration *C*_a_ = 2.55% which corresponds to the assumed elastic modulus of the bacteria *E*_b_ = 375 kPa. Note that this also coincides with *C*_a_ = 2.5% at which we observe a peak in the colony size at invasion in our experiments. (*c*) The density of the bacteria in the colony shows no dependence on the radial position. (*d*) The mean density of bacteria in the colony increases with agarose concentration. Images correspond to colonies at agarose concentrations of 1.5% (left) and 3.5% (right). The number of colonies analysed for (*a,b*) is between nine and 24 for each agarose concentration, whereas for (*c,d*) between six and 15 colonies are analysed for each agarose concentration. There are typically 250 bacteria in the colonies at 1.5% when invasion occurs (approx. eight generations) whereas for 3% there are typically 500 bacteria (approx. nine generations). Simulation results presented in all panels have been averaged over 50 independent runs, and error bars represent standard errors of the mean. (Online version in colour.)

The microcolonies in our experiments are roughly circular-symmetric; the eccentricity at the onset of invasion is shown in figure 2*f* (note that eccentricity is defined as *e* = (1 − *a*^2^/*b*^2^)^0.5^, where *a*,*b* are the minor, major axes of the ellipse, and values around 0.5 correspond to ellipses which are still very circular). We observe that invasion of the agarose by the bacteria consistently starts close to the centre of the microcolony. Using confocal microscopy, we were able to pinpoint the position **x**_inv_ within the microcolony where the first bacterium escapes from the two-dimensional microcolony to form a second vertical layer. In order to compare microcolonies of different sizes, we define the dimensionless distance3.1
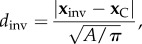
where **x**_C_ is the centre of mass of the microcolony and *A* is its area. Thus, *d*_inv_ = 0 if invasion happens at the centre of the microcolony, and *d*_inv_ = 1 if it happens at the very edge of the microcolony. Our results show that invasion always occurs near the centre of the microcolony, with *d*_inv_ ≈ 0.2 (figure 3*a*), over a wide range of agarose concentrations.

#### 3.1.3. Invasion does not require secreted factors

**Figure 4.**
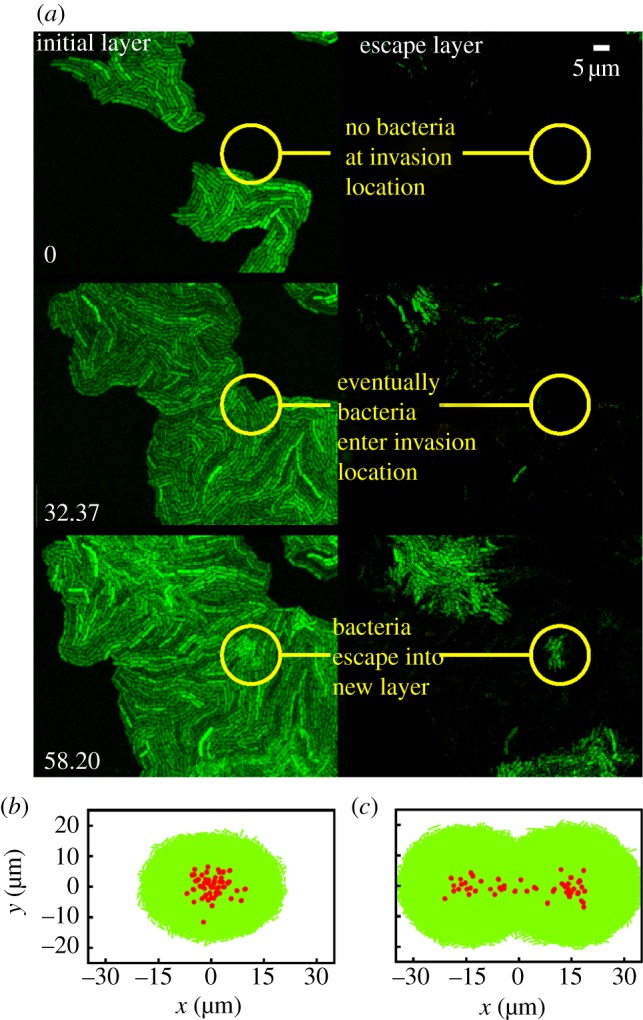
Invasion does not require chemical factors. (*a*) Colonies that collide as they grow are shown at three different times (as labelled min.s), and at two focal planes separated by 2.1 µm. At the time of the first image, some initially separated colonies have already collided, but bacterial escape has not yet occurred anywhere. As the collision proceeds, the second layer of cells begins to form at a point denoted by the yellow circle. Invasion thus occurs in the area previously not occupied by bacteria. This implies that invasion is not triggered by chemical cues, because chemical factors secreted by the bacteria could not have accumulated at the collision site. (*b,c*) Simulations of pairs of colliding colonies. Colonies were seeded from pairs of cells initially located (*b*) 10 µm and (*c*) 30 µm apart, and simulations were run until the moment of the invasion transition. The figures show overlays of the results of 50 simulations; the positions of the bacteria at the moment of invasion are shown as green rods, whereas the position at which the invasion transition happens is shown by the red dots. Invasion often (but not always) occurs close to the point where the colonies collide (0,0), rather than at the original centres of the two microcolonies.

The fact that invasion occurs near the colony centre might suggest that it is triggered by biochemical factors secreted by the cells, which might affect their mechanical behaviour (for example secreted factors can affect motility in *Legionella pneumophila* [27] and *Pseudomonas aeruginosa* [28]), or the physical structure of the agarose [29]. Such factors would be expected to be present at the highest concentration, and for the longest time, near the microcolony centre. To test this hypothesis, we tracked the invasion transition in *colliding microcolonies*—i.e. microcolonies that originate from closely spaced cells and collide as they expand in two dimension. In these experiments, we would still expect accumulation of secreted factors near the centres of the original individual microcolonies. Importantly, however, we observed that invasion of the agarose often occurred at the contact point between colliding colonies, rather than at their original centres (figure 4*a*). This suggests that secreted biochemical factors are unlikely to be playing an important role in the invasion transition.

#### 3.1.4. The invasion transition shows a complex dependence on agarose concentration

To probe the mechanical forces acting within the microcolonies and their role in the invasion transition, we manipulated the elasticity of the agarose by varying its concentration. The elastic modulus of a very similar type of agarose to that used in our experiments (Sigma-Aldrich, A9539 agarose) has been experimentally determined in [26] (see the supplementary material therein) and can be described by3.2
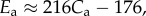
where *C*_a_ is the agarose concentration in wt% and *E*_a_ is the elastic modulus in kPa. Because our experiments were conducted for *C*_a_ = 1.5*–*4%, we expect that the elastic modulus *E*_a_ of the agarose in our experiments ranges from ≈150 to ≈700 kPa.^2^

Focusing first on the properties of the microcolonies prior to the invasion transition, we find that increasing the agarose concentration has no effect on the pre-invasion growth rate (figure 2*f*), but does increase the average density of cells within the growing colony (figure 3*d*). For all agarose concentrations, the cell density is rather uniform throughout the colonies (figure 3*c*).

Interestingly, the microcolony size at which the invasion transition happens shows a complex dependence on the agarose elasticity: for low agarose concentrations, the colony area at invasion increases approximately linearly with *C*_a_, but for higher agarose concentrations *C*_a_, the area at invasion actually *decreases* as *C*_a_ increases (figure 3*b*). The peak in the area at invasion occurs at an agarose concentration of *C*_a_ ≈ 2.5 wt%, corresponding to an elastic modulus *E*_a_ ≈ 360 kPa.

### 3.2. Mechanical theory

Our experimental observations can be understood by considering the nature of the forces acting on the bacteria within the microcolony. We assume that these forces consist of (i) repulsive forces between the bacteria (ii) frictional forces between neighbouring bacteria, and between bacteria and the glass and agarose surfaces, and (iii) elastic forces exerted by the agarose on the bacteria owing to its compression by the microcolony as it grows. In §3.3, we show that individual-based computer simulations, including these force contributions can account for the phenomena observed in our experiments. First, however, we consider in detail the nature of the elastic and frictional forces arising from the interactions between the microcolony and the agarose, and their implications for how the microcolony area at the transition should scale with the agarose concentration.

#### 3.2.1. Forces owing to compression of the agarose

As illustrated in figure 1, the growing microcolony compresses the agarose layer, which will therefore exert elastic forces on the bacteria. To calculate these forces, we assume that the microcolony can be treated as a rigid disc of radius *R* which is pressed into the agarose to a depth *d* ≈ 1.4 μm (this being a typical cell diameter in our experiments); this assumption is justified in appendix G. From the theory of contact mechanics [21], the vertical compression force per unit area *f_z_*(*r*) (figure 1), at a distance *r* from the centre of the microcolony, is3.3
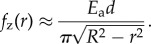
It is important to note that equation (3.3) diverges at *r* = *R*. This divergence is avoided in our simulations by using a cut-off (see Methods). We also expect there to be a radial contribution to the compression force acting on bacteria at the edge of the microcolony (figure 1). In appendix A, we show that the magnitude of this force (per unit area) is3.4
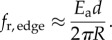
The quantities *f_z_* and *f*_r,edge_ are in units of kPa and can be thought of as normal stresses specified at the boundary of the colony.

#### 3.2.2. Forces owing to friction with the agarose

We also expect frictional forces, acting in the inward radial direction, to exist between the growing microcolony and the agarose and glass surfaces (figure 1), with magnitude proportional to the vertical compression stress *f_z_*. Previous observations of the surface friction of polymer gels [30] suggest that the friction force per unit area *f*_r_(*r*) can be generically expressed as3.5
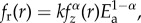
where *α* is an exponent that depends on the chemical structure of the gel (0 < *α* < 1), and *k* is a dimensionless friction coefficient that we expect to depend on the concentration of the agarose (note that in the case where *k* is a constant and *α* = 1, equation (3.5) reduces to Amonton's first law of friction *f*_r_ = *kf_z_*, or *F* = *kN* [31]).

In our experiments, we believe that this radial friction force plays a more important role than the radial elastic force that arises directly from compression of the agarose (i.e. 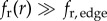). This is because, in our experiments with isolated microcolonies, invasion of the agarose occurs very close to the microcolony centre (figure 3*a*). We find that this phenomenon is very well reproduced in computer simulations where the radial elastic force is much smaller than the frictional forces. By contrast, when we make the frictional forces in our simulations much smaller than the radial elastic force, we see that invasion occurs further from the centre (see appendix D and figure 6), which is incompatible with the experimental data.

#### 3.2.3. Expected consequences for the friction coefficient

Building on the preceding theoretical arguments for the nature of the elastic and frictional forces, we now speculate briefly on the likely dependence of the friction coefficient *k* on the agarose concentration.

For the sake of simplicity, let us consider a one-dimensional analogue of a microcolony: a chain of *n* bacteria sandwiched between an agarose surface and a rigid substrate. We assume that the first bacterium is fixed at *x* = 0, so that as the bacteria proliferate, the chain extends along the positive semi-axis. The bacteria are compressed vertically with force per unit area 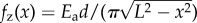, in analogy with equation (3.3) (where *L* is the length of the chain). The transition happens when the chain of bacteria ‘buckles’ in the vertical direction; we expect that this occurs, because small inhomogeneities translate inward horizontal forces within the chain into vertical forces. Once these become large enough to overcome the vertical compression force, the bacteria invade the agarose.

As the bacteria proliferate, the chain expands in the positive *x*-direction, and the bacteria experience frictional forces acting in the opposite direction. These forces will be transmitted along the chain, so that the maximal horizontal stress is experienced by the first bacterium in the chain (at *x* = 0). We can calculate the total horizontal force on this bacterium as3.6
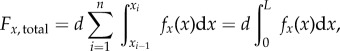
where 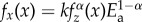 is the frictional force per unit area (by analogy with equation (3.5)), {*x_i_*} are the positions of the contact points between the bacteria, and *d* is the width of a bacterium.

This force is transformed into a vertical force pushing the bacteria into the agarose, by factors such as irregularities in the agarose (or glass) surface, differences in the diameters of individual bacteria or Euler buckling of individual bacteria. We represent this by supposing a vertical force component *aF_x_*_,total_, where 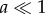 represents the factors that transform the horizontal force into vertical force, such as irregularities in the agarose surface or bacterial diameters.

The bacterium at *x* = 0 penetrates the agarose if this vertical force component, directed into the agarose, is greater than the vertical compression force (which is directed away from the agarose). This condition, together with equation (3.6) (inserting the form of *f_z_* and integrating), leads to the following expression for the critical chain length *L* at which the transition happens:3.7
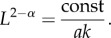
In equation (3.7), the explicit dependence on the elastic modulus of the agarose *E*_a_ has cancelled out. Thus, if we observe that the critical chain length *L* increases as the agarose concentration *C*_a_ increases, then this implies that either the factor *a* or the friction coefficient *k* must decrease as *C*_a_ increases. We expect that, in fact, *a* is either independent of *C*_a_ (if irregularities are caused by variations in the diameter of individual bacteria) or decreases with *C*_a_ (if irregularities are due to submicrometre-sized pores in the agarose surface [32]). This therefore implies that an increase in *L* with agarose concentration must be caused by a decrease in the friction coefficient *k*.

In our experiments, microcolonies are of course two-dimensional and it is the area of the colony at the transition which is observed to increase with the agarose concentration, at least for low agarose concentrations. While it is possible to construct a similar argument for the radial frictional stress field inside a circular microcolony, it is not clear whether the translation of horizontal into vertical stress can be represented in such a simple way. Nevertheless, our arguments for the one-dimensional bacterial chain do suggest that the strong dependence of the critical microcolony area on the agarose concentration which we observe in our experiments is likely to originate from a concentration-dependence of the friction coefficient.

### 3.3. Computer simulations

To demonstrate that mechanical arguments can, indeed, explain the full range of phenomena that we see in our experiments, we carried out individual-based computer simulations, in which elastic, rod-shaped bacteria grow, divide and interact mechanically with each other and with the agarose and glass surfaces. Here, we assume a Hertzian form for the contact interactions (see Methods and equation (F 1)), but similar results are obtained for other models, as shown in appendix F. The agarose is represented implicitly by position-dependent vertical and horizontal forces acting on the bacteria, as predicted by our theoretical arguments (equations (3.3)–(3.5); see Methods for details of the implementation).

**Figure 5.**
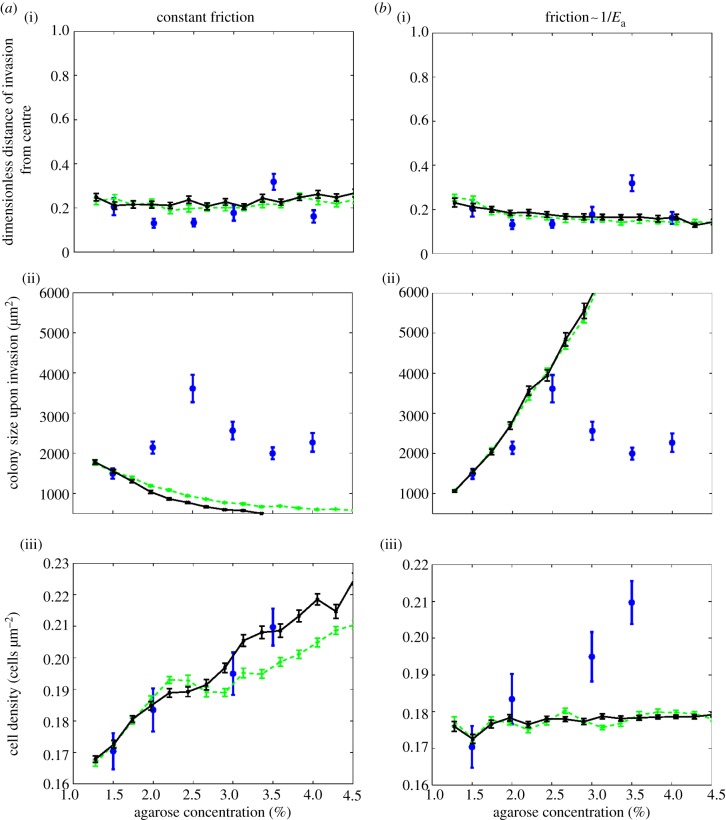
Changing the dependence of the friction coefficient on the agarose concentration affects whether the simulations match the experimental data. In all figures, solid lines correspond to *α* = 1 and dashed lines to *α* = 0.4 in equation (3.5). Circles show experimental data. *a*(i–iii) Simulations with *constant friction coefficient* (*k* = 0.7). The panels show (i) the dimensionless buckling distance, which matches the experiments well. (ii) The colony area upon invasion, which does not match the experimental data. (iii) The cell density at the transition, which also matches well. *b*(i–iii) Simulations with a *friction coefficient that is inversely proportional to the agarose concentration* (*k* = 0.7 × 148/*E*_a_). The panels show (i) the dimensionless buckling distance, which again matches well. (ii) The colony area upon invasion, which matches well up to 2.5%. (iii) The cell density, which does not match the experimental data. To fully match the experimental data, we require the friction coefficient to depend on the agarose concentration as described in equation (3.8). (Online version in colour.)

**Figure 6.**
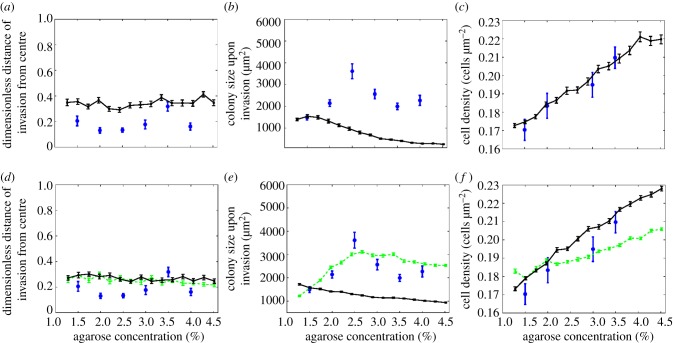
(*a*–*c*) Our experimental data are not reproduced by simulations in which boundary-induced compression of the colony by the agarose dominates over friction. The circles show our experimental data, whereas the solid lines show the results of computer simulations in which radial compression caused by the agarose acting on peripheral cells dominates friction from the substrate. Apart from the density of the bacteria at the transition (*c*), which agrees with the experimental data for *E*_b_ = 750 kPa, neither the area upon invasion (*b*) nor the reduced distance (*a*) can reproduce the corresponding data. This suggests that in our experiments, radial compression due to the agarose is less important than radial friction. (*d*–*f*) ‘Stokesian’ friction proportional to velocity leads to similar results as velocity-independent friction. The microcolony area upon invasion (*e*), reduced distance (*d*) and cell density upon invasion (*f*) are shown for constant *κ* (solid line) and *κ* depending on *C*_a_ as in equation (E 1) (dashed line). The circles show our experimental data. (Online version in colour.)

Most parameters in our simulation are fixed either by our own measurements or by literature values (see Methods). We determine the two remaining parameters, the elastic modulus of the bacteria *E*_b_, and the value of the friction coefficient *k*(*C*_a_) at one specific agarose concentration, by comparing our simulations with the experimentally determined area and cell density at the transition. We choose 1.5% agarose as the reference point; this corresponds to *E*_a_ = 148 kPa [26]. Performing simulations for many different values of *E*_b_ and *k*(*C*_a_ = 1.5%), we find that the microcolony area and cell density at the transition match those determined experimentally for 1.5 wt% agarose if we assume *E*_b_ = 375 kPa, and *k*(*C*_a_ = 1.5%) ≡ *k*_1.5%_ = 0.7 (figures 3 and 5). Interestingly, for this choice of parameters, we find that the reduced distance from the centre at which the transition happens also matches the experimental data quite well. It is important to note that although our assumed value *E*_b_ = 375 kPa for the bacterial elastic modulus is close to that of the agarose in our experiments, this does not imply that the bacteria are significantly deformed. In fact, equation (3.3) shows that for 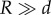 the compression force per unit area of microcolony, *f_z_*, is much smaller than *E*_a_ everywhere in the colony, except perhaps in a narrow ring close to the colony boundary.

The one-dimensional theory developed in §3.2.3 suggests that, in order to reproduce the non-monotonic dependence of the colony area at the transition on the agarose concentration *C*_a_, we are likely to need a friction coefficient *k* that depends on *C*_a_. Indeed, for simulations where we assume a constant value of *k*, we find that the area at the transition actually decreases slowly with *C*_a_ (see appendix B).^3^

It is therefore necessary to find a functional form of *k*(*C*_a_) that can match our experimental data for all values of the agarose concentration *C*_a_—i.e. we would like to find *k*(*C*_a_) such that the colony area at the transition first increases linearly with *C*_a_, reaches a peak at about 2.5% agarose, and then decreases again for larger concentrations.

Let us first consider the behaviour for small agarose concentrations. In appendix B, we show that good agreement with our data for concentrations up to 2.5% can be obtained if we assume *k*(*C*_a_) = *k*_1.5%_ × [148/*E*_a_(*C*_a_)], where *E*_a_(*C*_a_) is expressed in kPa (via equation (3.2)) and *k*_1.5%_ = 0.7 is the friction coefficient at *C*_a_ = 1.5%, obtained as discussed above.

For higher agarose concentrations, the area at the transition decreases with *C*_a_. To reproduce this behaviour, we assume that the friction coefficient *k*(*C*_a_) becomes constant for agarose concentrations above *C*_a_ = 2.5%, which corresponds to *E*_a_ = 364 kPa. Thus, the dependence of the friction coefficient on the agarose concentration which allows us to reproduce our experimental data is3.8
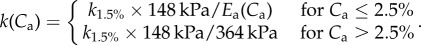
This functional form of the friction coefficient provides good agreement between the simulated and experimentally determined area at the transition (figure 3*b*). Moreover, for the same set of parameters, our simulation results also agree remarkably well with the experimental data for the distance from the centre of the colony at which invasion happens over the full range of agarose concentrations (figure 3*a*), and reproduce the experimental trend in the density of the bacteria as a function of agarose concentration (figure 3*d*)—although here the quantitative agreement is slightly less good. These results are not sensitive to the detailed form of the frictional forces: repeating our simulations for two different values of *α* (see equation (3.5)); *α* = 1 (Amonton's law of friction) and *α* = 0.4—the value found experimentally for agarose in contact with glass [30], we find that both choices produce almost identical results (figure 3).

It is important to note that the formula (3.8) should not be taken as a quantitative prediction for the dependence of the frictional forces on the agarose concentration; rather, it indicates only the qualitative trend in the friction coefficient that is necessary to match our experimental observations. While further experiments are needed to measure the friction coefficient of a bacterial cell on an agarose surface, such a non-monotonic dependence on the agarose concentration would not be entirely unexpected, because the friction coefficient between polymer gels and macroscopic surfaces has been observed to vary significantly with applied load (increasing or decreasing, depending on the gel type and its degree of swelling), and to depend in a non-trivial way on the polymer concentration [30]. In our experiments, mechanisms that might lead to such a dependence of the friction coefficient on the agarose concentration include changes in the water-mediated lubrication of the agarose surface with concentration, concentration-dependence of the pore size, making the agarose surface smoother at higher concentration, or compression of the bacteria, increasing their contact area with the gel/glass surface.

Using these parameters, we have also performed simulations in which two microcolonies grow close together in space, such that they collide prior to the invasion transition as in the experiments of figure 4*a* (see appendix C for details of how the elastic forces were implemented in this case). In these simulations, we observe that invasion often occurs at the contact point between colliding colonies, rather than at their original centres (figure 4*b*,*c*)—in agreement with the experimental results of figure 4*a*.

## 4. Discussion

Bacterial microcolonies provide attractive model systems for the study of multicellular assembly processes, because their development can be tracked in the laboratory at the level of individual cells, they are easy to manipulate (both physically and genetically) and, at least for *E. coli*, relatively few factors are at play. As well as mimicking eukaryotic assembly processes such as the development of animal organs or tumour growth, bacterial communities provide an important test system for investigating the role of spatial structure in evolutionary processes such as the evolution of drug resistance, both in bacteria and in cancer [33,34]. Understanding microcolony growth also provides direct insights into the processes at play in the early stages of bacterial biofilm formation and development.

Here, we have investigated the two- to three-dimensional transition that happens when an *E. coli* microcolony invades a soft agarose surface. This transition provides a sensitive probe of the interactions both within the microcolony and with its environment, and also mimics clinically and industrially relevant situations such as the invasion of soft tissue by bacteria growing on the surface of a medical implant or catheter, and the invasion of foodstuff by pathogenic bacteria.

Our experimental results depend in a complex way on the elasticity of the agarose; the density of cells within the microcolony increases with the agarose concentration, whereas the colony area at which the two- to three-dimensional transition happens peaks at an intermediate agarose concentration. Using phenomenological theory, we show that the compression of the agarose by the microcolony and frictional forces between the bacteria and the agarose both play key roles. Combining this theory with individual-based computer simulations, we show that mechanical interactions can explain all the observed features of colony growth, and of the two- to three-dimensional transition, observed in our experiments. The essential ingredients that we need in our simulations to reproduce the experimental data are (i) vertical and radial compression forces proportional to the elastic modulus of the agarose *E*_a_; (ii) a friction coefficient which decreases for low agarose concentrations *C*_a_ and reaches an asymptotic value at higher agarose concentrations; (iii) a mechanism by which the cell packing can increase upon compression (in our simulations, this can happen either by bending or by longitudinal or transverse compression of the cells). We do not need to include any biochemical factors to reproduce our experimental results; indeed, these appear to be ruled out to some extent by our experimental observations for colliding colonies.

Recently, individual-based computer simulations, in which rigid, rod-shaped bacteria interact via simple repulsive forces, have been successful in reproducing several features of the morphology and dynamics of bacterial communities [23–25,35]. Our simulations go a step further by including the elasticity of the bacterial cells and the elastic interactions between the bacteria and the surrounding agarose medium. These features turn out to be essential in explaining the key features of the two- to three-dimensional transition, suggesting that elastic forces may also play an important role in other aspects of bacterial colony morphology. Our results also highlight the key role of frictional forces, which are often treated only crudely in individual-based models, and have not, so far, been studied experimentally in any detail. Our analysis allows us to make a non-trivial qualitative prediction about the concentration-dependence of the friction coefficient between an *E. coli* bacterium and agarose and glass surfaces, which should be testable using existing optical tweezers methods.

In our analysis, we have treated the agarose as an elastic medium, neglecting plastic deformations (i.e. breakage of the agarose gel structure). Although such plastic deformations may play a role for small colonies, where the vertical compression force *f_z_*(*r*) within the microcolony can become larger than the compressive strength of agarose (http://www.sigmaaldrich.com/content/dam/sigma-aldrich/docs/Sigma/Product_Information_Sheet/a9539pis.pdf (approx. 100 kPa for *C*_a_ = 1%), this is not likely to be the case for colonies of size *R* > 10 μm, for which *f_z_*(*r*) remains below the compressive strength of agarose everywhere in the colony (for example, *f_z_*(0) = 5 kPa for *C*_a_ = 1.5%).

A key unknown parameter in our simulations is *E*_b_, the effective elastic modulus of a bacterial cell. The best match between our simulations and the experimental data is obtained for *E*_b_ = 375 kPa. This is in fact in agreement with a value obtained recently in a different experiment by Tuson *et al.* [26]. These authors measured the elastic modulus of the *E. coli* cell wall to be *E*_wall_ = 50–150 MPa. Assuming that the cell can be modelled by an empty cylinder of diameter *d* = 1.4 μm and wall thickness *h* = 4 nm, one can compute the effective Young modulus of the bacterial cell in the longitudinal direction as *E*_b_ ≈ 2*hE*_wall_/*d* = 285−860 kPa; our fitted value falls within this range. This calculation neglects turgor pressure of about 30 kPa [36] present in live *E. coli* cells; this is justified *a posteriori*, because the computed value of *E*_b_ is much larger than 30 kPa.

While plausible, an agarose concentration-dependent friction coefficient is not the only possible explanation of the non-monotonic dependence of the area upon transition on the agarose concentration which we observe in our experiments. Another factor that could be important is adhesion of cells to the glass slide—a phenomenon that is known to create multi-nN forces in *B. thuringiensis* spores [37]. Preliminary simulations (not shown) suggest that a constant (agarose concentration-independent) radial force contribution owing to adhesive forces could conceivably generate an increasing dependence of the transition area with the agarose concentration and a decrease of the apparent friction coefficient *k*, but only if there is no corresponding vertical component to the adhesive force; however, to test this hypothesis, detailed measurements of adhesive forces between *E. coli* cells and glass and agarose surfaces would be needed. More generally, our work highlights the urgent need for more single-cell measurements of the mechanical properties of bacteria, and the mechanical interactions between bacteria and their environment. In particular, micro-indentation atomic force microscope measurements of elastic and frictional forces between bacteria and agarose would be extremely valuable.

## 5. Conclusion

Taken together, our experimental data and simulation results convincingly point to the importance of mechanics in the invasion transition of the growing bacterium colony, as opposed to hypothetical biochemical effects acting on the bacteria or on the agarose structure. In future work, we will explore in greater detail the transmission of force through the colony, which should be experimentally accessible by tracking tracer particles in the agar, as is routinely done in tracking forces generated by cellular tissues [38]. It would also pose stringent tests on the mechanical model if it were possible to measure micromechanical parameters such as the coefficient of friction experienced by individual bacteria in the conditions of these experiments.

The transition to bulk growth in *E. coli* microcolonies is a significant limitation in microscopic studies of single-cell physiology and gene regulation as well as limiting potential designs for bacteria-based biosensors. Our results show that this transition cannot easily be prevented by changing the elasticity of the gel material. More broadly, this work suggests that invasion of a soft material, whether a foodstuff or an animal tissue, by a growing mass of cells, whether a bacterial colony or a cancer tumour, is likely to depend in a highly non-trivial way on the elastic properties of the material being invaded.

The colony growth observed in this study embodies some aspects of biofilm growth, and maybe also other cell growth situations (e.g. colonization of plant or animal tissue). While here confinement was provided in a controlled manner, in other situations of cell growth, it can be more spatially complex, or can be an emergent property of the cell assembly itself. A next step of work, with specific challenges and questions, will be to perform experiments with different strains or species, possibly biofilm forming, to include explicit polysaccharide production and other structural features.

## Figures and Tables

**Figure 7.**
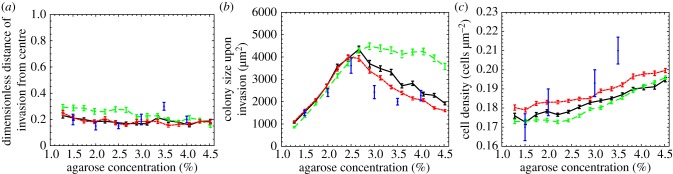
(*a*) Plots of the reduced distance of the position of invasion from the colony centre, (*b*) the microcolony area at the transition (*c*) and cell density at the invasion transition, for three different forms of the repulsion force: Hertzian (equation (F 1), solid line) with *E*_b_ = 375 kPa; equation (F 2) with *β* = 1, *E*_b_ = 200 kPa, *k*_1.5%_ = 0.7 (dashed line) and *β* = 2, *E*_b_ = 600 kPa, *k*_1.5%_ = 0.7 (dotted line). Unconnected markers are the experimental data. (Online version in colour.)

**Figure 8.**
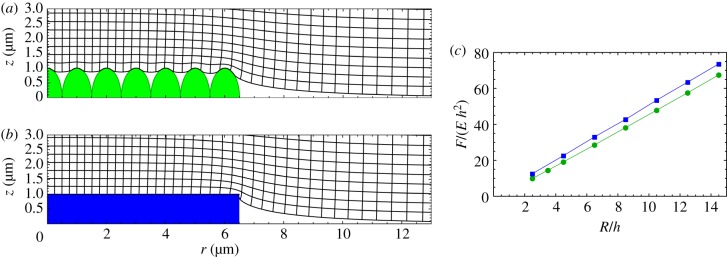
Bacterial colony modelled as a rigid punch indenting an elastic medium. (*a,b*) Distortion of the elastic medium (agarose) caused by the patterned punch (*a*) and the flat punch (*b*). The agarose does not penetrate the gaps between the grooves. (*c*) Total force exerted on the punch by the elastic medium for the two cases, as a function of the punch radius *R*. The patterning has little effect on the force. *h* is the height of the punch and *E* is the Young modulus of the elastic medium. All results were obtained numerically as described in the text. (Online version in colour.)

## Endnotes

1 In reality, *E*_glass_ is 10^3^–10^5^ times larger than *E*_b_, but this would require impractically small time steps in the numerical algorithm. Setting *E*_glass_ = 10 MPa as in our simulations prevents bacteria from invading the glass slide and we therefore consider this value large enough to capture the essential physics of the interaction.

2 The exact relationship between *E*_a_ and *C*_a_ can depend on the agarose type, preparation method, etc. The agarose used in our experiment was very similar to that of in [26] and, hence, we expect that equation (3.2) holds to a good approximation.

3 The fact that the area at the transition decreases with *C*_a_ in this case, rather than being constant, is probably caused by a more complicated distribution of the forces in the two-dimensional colony when compared with the one-dimensional case, and by the compression and bending of cells.

## Appendix A. Radial contribution owing to the agarose elasticity

Here, we show that the radial compression force acting on bacteria at the edge of the microcolony is approximately *E*_a_*d*/(2π*R*). As in the main text, we model the microcolony as a rigid disc, pressed vertically into the agarose. The energy required in this process is, according to equation (3.3),

A1

Let us now imagine, that, instead of pressing the disc of constant radius into the agarose, we first press an infinitesimally thin cylinder to depth *d* and then expand it from radius zero to radius *R*. The energy required for this process is

A2

where *f*_r,edge_ is the radial compression force per unit area, acting at the boundary and opposing the expansion. By equating (A 1) and (A 2) and differentiating with respect to *R*, we obtain that *f*_r,edge_(*R*) = *E*_a_*d*/(2π*R*).

## Appendix B. Constant friction coefficient *k*, or *k* ∼ 1/*E*_a_, do not reproduce our experimental data

Here, we show that if we assume either a constant friction coefficient *k*, or a friction coefficient that decreases with the agarose elastic modulus as *k* ∼ 1/*E*_a_, then our simulations do not reproduce our experimental data. In figure 5*a*, we present results of our simulations for *k* = const = 0.7, with the other simulation parameters chosen, so that the microcolony area upon invasion agrees with the experimental result for 1.5% agarose. Although both the dimensionless distance of the buckling point from the centre and the cell density at the transition agree well with the data, the microcolony area at the transition decays monotonically with increasing agarose concentration, failing to reproduce the peak seen in the experiments. The situation is slightly better for *k* = 0.7 × 148 kPa/*E*_a_, figure 5*b*, for which the area at the transition matches the experimental data up to the peak, but deviates from it for more than 2.5% agarose. These results clearly show that the form of equation (3.8), in which the friction coefficient initially decreases with increasing agarose concentration, and then plateaus for larger agarose concentrations, is needed to reproduce our experimental results.

## Appendix C. Simulations of colliding colonies

In figure 4*b*,*c*, we show the simulation of the growth of two colonies starting from individual cells that are initially separated by a distance of 10 µm (*b*) or 30 µm (*c*) using the same method described in the main text, with the only modification being the calculation of the vertical and radial compression forces. Instead of approximating the colony by a rigid disc with radius *R*, we use two discs of radii *R*_1_ and *R*_2_, centred at the initial positions of the two founder cells and having same areas as the two growing colonies. A bacterium is assumed to belong to disc 1 if its distance from the centre of disc 1 is smaller than from disc 2, and vice versa, and the area of the part of the colony that belongs to each disc is used to calculate the corresponding radius *R*_1_ or *R*_2_. Each bacterium experiences vertical compression given by equation (3.3), with *R* replaced either by *R*_1_ or *R*_2_, depending on whether the bacterium is closer to the centre of disc 1 or 2. The radial compression force acting on the boundary of each subcolony is calculated accordingly (i.e. using equation (3.4) but for two colonies). This force does not act on the bacteria that have already collided.

## Appendix D. Testing the relative importance of radial compression and radial frictional forces

In the main text, we hypothesized that the radial frictional forces *f*_r_(*r*) dominate the inward radial compression forces *f*_r,edge_ in our microcolonies. To test this hypothesis, we performed simulations for the case where the inward radial compression force was larger than the radial frictional forces, by using a smaller friction coefficient *k* = 0.1, and a larger radial compression *f*_r,edge_ (increased by a factor of 10 compared with our standard simulations). With this parameter set, the radial compression force was almost two orders of magnitude stronger than the friction force measured at the colony centre. Figure 6*a*–*c* shows that, in this case, the dimensionless distance of invasion becomes almost twice as large as it is in the experiments. This suggests that radial compression does not dominate in our system. Note that here we used an *E*_b_ = 750 kPa, different from elsewhere, to fix the area at *C*_a_ = 1.5%.

## Appendix E. Velocity-dependent friction has little effect on our results

We have also carried out simulations in which we assumed the friction coefficient to be proportional to the cells' velocity along the agar surface, *k* = *κv*, with proportionality coefficient *κ*. In these simulations, we either assumed that *κ* was constant, or that it depended non-monotonically on the agarose concentration *C*_a_ in the same way as for the static friction coefficient *k* in our previous simulations (i.e. via an equation similar to equation (3.8)):

E1

where *κ*_1.5%_ = 0.05 was chosen to fix the area and distance upon transition at *C*_a_ = 1.5% to experimentally determined values. Figure 6*d*–*f* shows that all three quantities that we are interested in (dimensionless distance of the transition point from the microcolony centre, microcolony area upon invasion and cell density at the transition) behave in a similar way as in our simulations with velocity-independent friction (compare with figures 3 and 5). The agreement with the experimental data is, however, slightly poorer than for the static friction.

## Appendix F. The form of the contact interaction force between bacteria

In the simulations presented in the main text (e.g. figure 3), we have assumed that bacterial cells interact via a Hertzian-like repulsive force

F1

where *r* = 0.7 μm is the radius of the spheres that make up our bacterial rods, *ν* is the Poisson ratio (not known, but assumed here to be *ν* ≈ 0) and *ε* is the overlap distance, equal to the diameter *d* minus the minimal distance between the centres of the two interacting spherical segments of the bacteria. Equation (F 1) assumes that the interacting segments behave as solid, elastic spheres with Young modulus *E*_b_.

We also carried out simulations of growing bacteria colonies for two force–field models other than the Hertzian model:

F2

for *β* = 1 and *β* = 2. Figure 7 shows that our results for *β* = 1 deviate slightly from the experimental data, but those for *β* = 2 reproduce our data almost as well as the Hertzian model. In both cases, to optimize the agreement with the data, we need to adjust slightly the elastic modulus *E*_b_ and the friction coefficient: (*E*_b_, *k*_1.5%_) = (200 kPa, 0.5) for *β* = 1, and (*E*_b_, *k*_1.5%_) = (600 kPa, 0.7) for *β* = 2. The reasonable agreement of both models with the data indicates that the actual relationship between the force and displacement does not affect our results very much, as long as *F* grows superlinearly with *ε*.

One may wonder however if this generic form is a good approximation for the interaction forces between bacterial cells, which are thin shells filled with cytoplasm, rather than homogeneous elastic bodies. The contact mechanics of elastic, empty shells has been extensively studied and various forms for *F*(*ε*) for interactions between spherical shells have been proposed [39–41]. Assuming the radius and thickness of the shell to be *r* and *h*, respectively, and the elastic modulus of the wall to be *E*_w_, theoretical models [39,40] suggest *F* ∝ *E*_w_(*h*/*r*)*ε*^2^ for small deformations (*ε* < *h*)). For large deformations, involving ‘buckling’ of the contact disc between two interacting surfaces for , in [39,40] give *F* ∝ *E*_w_(*h*^5/2^/*r*)*ε*^1/2^. However, numerical simulations and experiments [39,42] generally favour the linear, or weakly superlinear form, *F* ∝ *ε*^*β*^ with , similar to the theoretical prediction for interacting cylinders without adhesion (see, e.g. fig. 3 from [43]). For cylindrical shells, simulations for empty cylinders which include adhesion [43] indicate superlinear behaviour in *ε*. Moreover, all these models deal with hollow objects, whereas a cytoplasm-filled *E. coli* cell has an internal turgor pressure of at least 30 kPa [36] which may affect its stiffness. Because detailed modelling of the bacterial cell wall is beyond the scope of this paper, and (as shown above) the exact form of the force does not strongly affect our results, we leave this problem for future investigation.

## Appendix G. Justifying the use of the rigid punch approximation

In our theory and simulations, we have computed the forces resulting from compression of the agarose by assuming that the microcolony behaves as a flat, rigid cylinder pressed into the agarose. This assumption leads to equation (3.3). To test this assumption, we here examine how the vertical compression force changes if, instead of assuming a flat punch, we allow the surface of the punch to be patterned as to resemble the non-uniform surface of the colony, i.e. the contours of individual bacterial cells. A particularly important question here is whether the agarose is expected to penetrate into the gaps between the bacterial cells. We consider a circular, axially symmetric punch of radius *R* whose surface height varies with the radial coordinate *r* as

G1

so that (assuming all distances are measured in µm) when the maximal height *h* = 1 μm, the surface is covered with half-ellipsoidal wrinkles of approximately 1 µm size (figure 8). Assuming cylindrical symmetry, the displacement field ***u*** = (*u_r_*(*r*, *z*), *u_z_*(*r*, *z*)) of the agarose obeys the following equilibrium equation, which can be derived from the corresponding equation in Cartesian coordinates given in references [21,44]:

G2

and

G3

where *a* = (1 − 2*ν*)/(2 − 2*ν*), *b* = 1/(2 − 2*ν*) and *ν* is the Poisson ratio of agarose (we take for the sake of simplicity *ν* = 0 so that *a* = *b* = 1/2). We assume the colony is small compared with the agarose slab (radius *r*_max_ height *z*_max_), so that the displacements are zero at the boundary of our domain: ***u***(*r*, *z*_max_) = ***u***(*r*_max_, *z*) = **0**. Cylindrical symmetry implies that ∂_*r*_*u*_*z*_(0, *z*) = 0 and *u_r_*(0, *z*) = 0. The punch cannot be penetrated by the elastic medium, so that

G4

We further assume infinite friction between the punch and the medium, so that *∂_r_u_r_*(*r*, 0) = 0 (we have checked that the case of zero friction leads to similar results).

As a convenient measure, we compute how the total force

G5

exerted on the punch by the elastic medium changes with the radius *R* of the punch.

We have solved equations (G 2) and (G 3) numerically, on a mesh with variable mesh size (the mesh size was chosen to be finer close to the colony where displacements were largest; the larger mesh size close to the boundaries allowed us to use a large domain *r*_max_, *z*_max_, avoiding boundary effects). Figure 8, left, shows the displacement field ***u*** of the agarose for a punch of height *h* = 1 μm and radius *R* = 6.5 μm, for the patterned punch (top) and a flat punch (bottom). Even though this punch radius is relatively small, implying large elastic forces, the agarose does not penetrate into the gaps between the bacterial cells for the patterned punch. Figure 8, right, further confirms this: in both cases the total force *F* varies linearly with the radius *R* of the punch, with almost identical slope. We also checked (results not shown) that the compression force from equation (3.3) for the flat punch provides a good approximation for the force exerted on each of the ‘cells’ of the patterned punch. Taken together, these results suggest that our assumption of a flat punch is indeed appropriate as a model for the bacterial colony.

## References

[1] Wolpert L 1998 Principles of development (eds Wolpert L Beddington R Brockes J Jessell T Lawrence P Meyerowitz E). Oxford, UK: Oxford University Press.

[2] Liotta LA Kohn EC 2001 The microenvironment of the tumour–host interface. Nature 411, 375–379. (10.1038/35077241) 11357145

[3] Mammoto T Ingber DE 2010 Mechanical control of tissue and organ development. Development 137, 1407–1420. (10.1242/dev.024166) 20388652 PMC2853843

[4] Marinari E Mehonic A Curran S Gale J Duke T Baum B 2012 Live-cell delamination counterbalances epithelial growth to limit tissue overcrowding. Nature 484, 542–545. (10.1038/nature10984) 22504180

[5] Coucouvanis E Martin GR 1995 Signals for death and survival: a two-step mechanism for cavitation in the vertebrate embryo. Cell 83, 279–287. (10.1016/0092-8674(95)90169-8) 7585945

[6] Gordon JI Hermiston ML 1994 Differentiation and self-renewal in the mouse gastrointestinal epithelium. Curr. Opin. Cell Biol. 6, 795–803. (10.1016/0955-0674(94)90047-7) 7880525

[7] Asally M 2012 Localized cell death focuses mechanical forces during 3D patterning in a biofilm. Proc. Natl Acad. Sci. USA 109, 18 891–18 896. (10.1073/pnas.1212429109) 23012477 PMC3503208

[8] Pera EM Ikeda A Eivers E Robertis EMD 2003 Integration of IGF, FGF, and anti-BMP signals via Smad1 phosphorylation in neural induction. Genes Dev. 17, 3023–3028. (10.1101/gad.1153603) 14701872 PMC305254

[9] Hodor PG Illies MR Broadley S Ettensohn CA 2000 Cell–substrate interactions during sea urchin gastrulation: migrating primary mesenchyme cells interact with and align extracellular matrix fibers that contain ECM3, a molecule with NG2-like and multiple calcium-binding domains. Dev. Biol. 222, 181–194. (10.1006/dbio.2000.9696) 10885756

[10] Ridley AJ 2003 Cell migration: integrating signals from front to back. Science 302, 1704–1709. (10.1126/science.1092053) 14657486

[11] Harris WA 1997 Pax-6: where to be conserved is not conservative. Proc. Natl Acad. Sci. USA 94, 2098–2100. (10.1073/pnas.94.6.2098) 9122153 PMC33656

[12] Martiny AC Jorgensen TM Albrechtsen HJ Arvin E Molin S 2003 Long-term succession of structure and diversity of a biofilm formed in a model drinking water distribution system. Appl. Environ. Microbiol. 69, 6899–6907. (10.1128/AEM.69.11.6899-6907.2003) 14602654 PMC262284

[13] Grice EA 2009 Topographical and temporal diversity of the human skin microbiome. Science 324, 1190–1192. (10.1126/science.1171700) 19478181 PMC2805064

[14] Carpentier B Cerf O 1993 Biofilms and their consequences, with particular reference to hygiene in the food industry. J. Appl. Bacteriol. 75, 499–511. (10.1111/j.1365-2672.1993.tb01587.x) 8294303

[15] Parsek MR Singh PK 2003 Bacterial biofilms: an emerging link to disease pathogenesis. Annu. Rev. Microbiol. 57, 677–701. (10.1146/annurev.micro.57.030502.090720) 14527295

[16] Stewart EJ Madden R Paul G Taddei F 2005 Aging and death in an organism that reproduces by morphologically symmetric division. PLoS Biol. 3, 295–300. (10.1371/journal.pbio.0030045) PMC546039 15685293

[17] Donlan RM Costerton JW 2002 Biofilms: survival mechanisms of clinically relevant microorganisms. Clin. Microbiol. Rev. 15, 167–193. (10.1128/CMR.15.2.167-193.2002) 11932229 PMC118068

[18] Henrichsen J 1972 Bacterial surface translocation: a survey and a classification. Bacteriol. Rev. 36, 478–503. 4631369 10.1128/br.36.4.478-503.1972 PMC408329

[19] Harshey RM 2003 Bacterial motility on a surface: many ways to a common goal. Annu. Rev. Microbiol. 57, 249–273. (10.1146/annurev.micro.57.030502.091014) 14527279

[20] Han SM Benaroya H Wei T 1999 Dynamics of transversely vibrating beams using four engineering theories. J. Sound Vib. 225, 935–988. (10.1006/jsvi.1999.2257)

[21] Landau LD Lifschitz EM 2008 Theory of elasticity, 3rd edn Oxford, UK: Elsevier.

[22] Hoffman H Frank ME 1965 Synchrony of division in clonal microcolonies of *Escherichia coli*. J. Bacteriol. 89, 513–517. 14255721 10.1128/jb.89.2.513-517.1965 PMC305535

[23] Volfson D Cookson S Hasty J Tsimring L 2008 Biomechanical ordering of dense cell populations. Proc. Natl Acad. Sci. USA 105, 15 346–15 351. (10.1073/pnas.0706805105) PMC2563119 18832176

[24] Boyer D Mather W Mondragón-Palomino O Orozco-Fuentes S Danino T Hasty J Tsimring LS 2011 Buckling instability in ordered bacterial colonies. Phys. Biol. 8, 026008 (10.1088/1478-3975/8/2/026008) 21358041 PMC3767764

[25] Rudge TJ Steiner PJ Phillips A Haseloff J 2012 Computational modeling of synthetic microbial biofilms. ACS Synth. Biol. 1, 345–352. (10.1021/sb300031n) 23651288

[26] Tuson HH 2012 Measuring the stiffness of bacterial cells from growth rates in hydrogels of tunable elasticity. Mol. Microbiol. 84, 874–891. (10.1111/j.1365-2958.2012.08063.x) 22548341 PMC3359400

[27] Stewart CR Rossier O Cianciotto NP 2009 Surface translocation by *Legionella pneumophila*: a form of sliding motility that is dependent upon type II protein secretion. J. Bacteriol. 191, 1537–1546. (10.1128/JB.01531-08) 19114479 PMC2648193

[28] Fauvart M Phillips P Bachaspatimayum D Verstraeten N Fransaer J Michiels J Vermant J 2012 Surface tension gradient control of bacterial swarming in colonies of *Pseudomonas aeruginosa*. Soft Matter 8, 70–76. (10.1039/c1sm06002c)

[29] Tipler LS Embery G 1985 Glycosaminoglycan-depolymerizing enzymes produced by anaerobic bacteria isolated from the human mouth. Arch. Oral Biol. 30, 391–396. (10.1016/0003-9969(85)90065-2) 3927877

[30] Gong J Osada Y 2002 Surface friction of polymer gels. Prog. Polym. Sci. 27, 3–38. (10.1016/S0079-6700(01)00037-5)

[31] Gao J Luedtke WD Gourdon D Ruths M Israelachvili JN Landman U 2004 Frictional forces and Amontons’ law: from the molecular to the macroscopic scale. J. Phys. Chem. B 108, 3410–3425. (10.1021/jp036362l)

[32] Narayanan J Jun-Ying X Xiang-Yang L 2006 Determination of agarose gel pore size: absorbance measurements *vis a vis* other techniques. J. Phys. Conf. Ser. 28, 83–86. (10.1088/1742-6596/28/1/017)

[33] Lambert G Estévez-Salmeron L Oh S Liao D Emerson BM Tlsty TD Austin RH 2011 An analogy between the evolution of drug resistance in bacterial communities and malignant tissues. Nat. Rev. Cancer 11, 375–382. (10.1038/nrc3039) 21508974 PMC3488437

[34] Greulich P Waclaw B Allen RJ 2012 Mutational pathway determines whether drug gradients accelerate evolution of drug-resistant cells. Phys. Rev. Lett. 109, 088101 (10.1103/PhysRevLett.109.088101) 23002776

[35] Farrell FDC Hallatschek O Marenduzzo D Waclaw B 2013 Mechanically driven growth of quasi-two dimensional microbial colonies. Phys. Rev. Lett. 111, 168101 (10.1103/PhysRevLett.111.168101) 24182305

[36] Deng Y Sun M Shaevitz JW 2011 Direct measurement of cell wall stress stiffening and turgor pressure in live bacterial cells. Phys. Rev. Lett. 107, 158101 (10.1103/PhysRevLett.107.158101) 22107320

[37] Kweon H Yiacoumi S Tsouris C 2011 Friction and adhesion forces of *Bacillus thuringiensis* spores on planar surfaces in atmospheric systems. Langmuir 27, 14 975–14 981. (10.1021/la203575q) 22059743

[38] Gjorevski N Nelson CM 2012 Mapping of mechanical strains and stresses around quiescent engineered three-dimensional epithelial tissues. Biophys. J. 103, 152–162. (10.1016/j.bpj.2012.05.048) 22828342 PMC3388222

[39] Pauchard L Pomeau Y Rica S 1997 Dformation Des Coques lastiques. C R Acad. Sci. IIB Mech. Phys. Chem. Astron. 324, 411–418.

[40] Pauchard L Rica S 1998 Contact and compression of elastic spherical shells: the physics of a ping–pong ball. Phil. Mag. B 78, 225–233 (10.1080/13642819808202945)

[41] Vaziri A 2009 Mechanics of highly deformed elastic shells. Thin-Walled Struct. 47, 692–700. (10.1016/j.tws.2008.11.009)

[42] Shorter R Smith JD Coveney VA Busfield JJC 2010 Axial compression of hollow elastic spheres. J. Mech. Mater. Struct. 5, 693–705. (10.2140/jomms.2010.5.693)

[43] Majidi C Wan KT 2010 Adhesion between thin cylindrical shells with parallel axes. J. Appl. Mech. 77, 041013 (10.1115/1.4000924)

[44] Johnson KL Johnson KL 1987 Contact mechanics. Cambridge, UK: Cambridge University Press.

